# Intact reinforcement learning in healthy ageing

**DOI:** 10.1007/s00221-025-07092-x

**Published:** 2025-07-11

**Authors:** Wei-Hsiang Lin, Karin S. Pilz, Michael H. Herzog, Marina Kunchulia

**Affiliations:** 1 Laboratory of Psychophysics, Brain Mind Institute, École Polytechnique Fédérale de Lausanne (EPFL), Lausanne, Switzerland; 2 Cito Institute for Educational Measurement, Arnhem, The Netherlands; 3 Institute of Cognitive Neurosciences, Free University of Tbilisi, Tbilisi, Georgia

**Keywords:** Reinforcement learning, Learning, Behavior, Ageing

## Abstract

What changes with age? Results in reinforcement learning (RL) are mixed. Some studies found deteriorated RL performance in older participants compared to younger controls whereas other studies did not. Daniel et al. (J Neurosci 40(5):1084–1096, 2020. 10.1523/JNEUROSCI.0254-19.2019) suggested that task demand can explain these differences, with less demanding tasks showing no effect of age. Compared to classic, simple RL tasks, we used a navigation task to increase overall complexity. We found that older adults performed less efficiently initially; however, with sufficient trials, they performed as well as young adults. Our results support the idea that ageing does not universally impair performance and that even higher-level cognitive tasks remain largely intact.

**Supplementary Information:**

The online version contains supplementary material available at 10.1007/s00221-025-07092-x.

## Introduction

To understand the mechanisms of ageing, it is crucial to first identify which functions decline and which remain stable. Learning is often thought to significantly diminish with age (Anguera et al. 2011; Salthouse 2009). A particularly interesting case of learning—and a typical one in both humans and animals is—reinforcement learning (RL), where observers learn through trial and error to reach a certain goal. A prominent example of RL is searching for food in an unfamiliar environment (e.g., flowers for honey bees or restaurants for humans). Here, an agent learns to navigate by exploring, adapting and making decisions based on feedback from its actions, gradually improving the strategy to locate food more efficiently. In experimental settings, animals explore mazes by deciding whether to go to the left or right arm at a crossing, also called a state. Entering into one arm is considered an action. Both animals and humans learn quickly to find the arms where food is provided by the experimenter (Kim et al. 2009; Tomov et al. 2021). These rewarding arms are referred to as goal states in RL. Powerful algorithms have been developed to explain the mechanisms underlying human and animal reinforcement learning (Watkins and Dayan 1992). The common idea behind all these algorithms is that when a goal state is found, the action leading to the goal state is reinforced and chosen the next time with a higher probability. Similarly, actions leading to the penultimate state are reinforced over time, allowing a successful path through the environment to be learned.

Computationally, RL differs from other types of learning, such as supervised learning. Whereas in reinforcement learning, feedback is often delayed and indirect, as rewards are given based on a sequence of actions rather than each individual action so that successful strategies are based on exploration and experimentation, in supervised learning, feedback is more explicit, direct, and immediate, mapping inputs such as certain actions to a particular immediate output.

Whether RL deteriorates in older adults is an open question. The use of similar paradigms has, in previous studies, led to different results and conclusions. In the most common paradigm used to investigate RL, an image is presented, and participants push one of two buttons to receive a positive, negative, or neutral reward. After the reward, the next image is presented randomly from a set of given images. Whereas Daniel et al. (2020) and van de Vijver and Ligneul (2020) found a clear age-difference using this tasks, others did not (Eppinger et al. 2008; Lighthall et al. 2018). However, the task in these studies is not very demanding, provoking only light deficits in both the young and old groups. For this reason, Daniel et al. (2020) have proposed that deficits of older people are only found in demanding tasks.

**Fig. 1:**
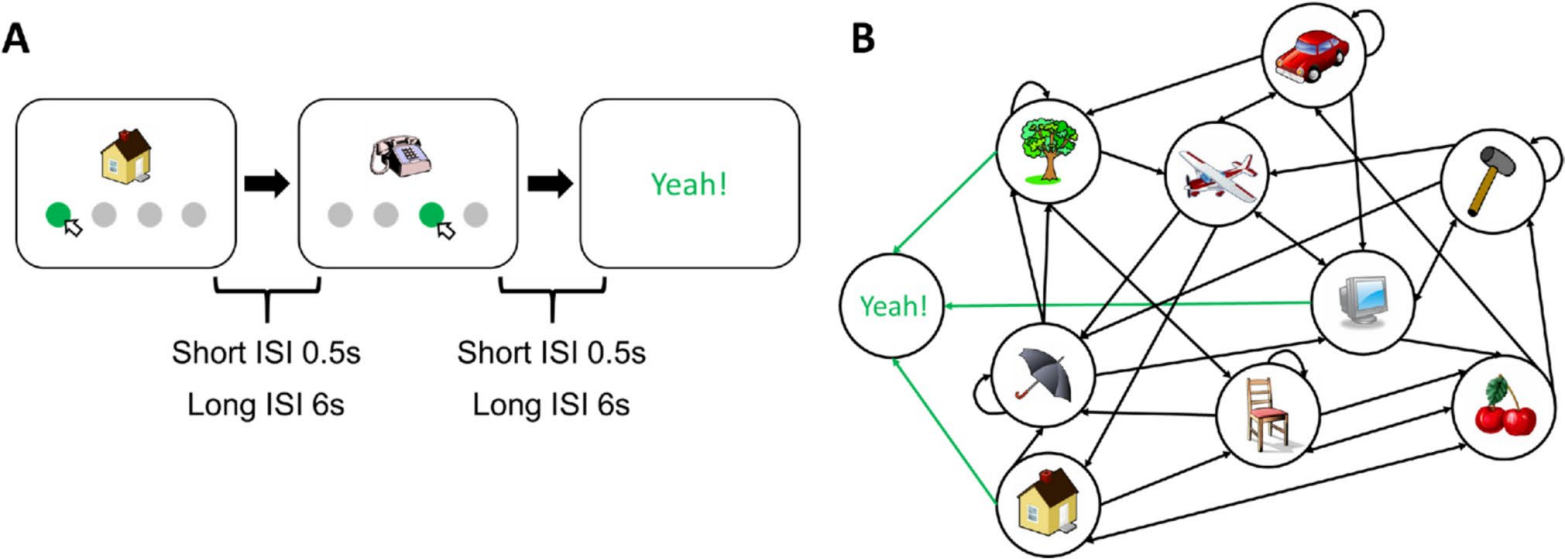
RL task. **A** A clip-art image appeared on the screen. Participants chose one of the disks below the image to proceed to the next image (state) until they found the goal state (“Yeah!”). Each participant performed under two different inter-stimulus interval (ISI) conditions, the short (0.5 s) and long (6 s). In experiment 1, participants had to reach the goal state as many times as possible within 8 min for the short ISI and 30 min for the long ISI condition. In experiment 2, they had to find as often as possible the goal image within 150 actions. **B** Structure of the RL environment (a visualization of the transition matrix). Each image represents a state, and the direction of the arrow indicates the connection between different states. Green arrows indicate the path leading to the goal state. Importantly, the structure of the environment is very irregular in the sense that observers may go directly from image **A** to image **B** but not necessarily back. We used irregular structures to prevent observers from using cognitive inference, which is possible, for example in grid-like environments. In all experiments, there are a total of nine states plus one goal state. As primary measures, we extracted 6 parameters including the number of episodes completed, the proportion of optimal actions, the improvement in both the number of episodes completed and the proportion of optimal actions, the learning rate, and exploration rate

Here, we used a more demanding paradigm, where the presentation of image *n* was not chosen randomly, as in the above studies, but depended on the choice made by the participants at image *n* − 1. One image was a goal image, and participants were asked to find it as often as possible within a certain period of time (Fig. 1). Hence, the experiment mimics a navigation task, which is more realistic than the above paradigms and involves additional aspects such as a systematic exploration of the RL environment, linking states not only to actions but also to other states. To independently test the effect of memory, we used not only a short (0.5 s) but also a longer (6 s) inter-stimulus intervals (ISI) between images. Various parameters were extracted from the participants’ performance and a Q-learning model was fitted. To preface our results, older participants initially exhibited less efficient learning. However, over the long term, their performance was comparable to that of younger controls.

## Materials and methods

### Experiment 1

#### Participants

Forty older healthy adults and thirty healthy young adults were recruited from the Free University of Tbilisi, Georgia. Detailed demographical information is presented in Table 1.

**Table 1:** The demographic information of participants

Experiment	Experiment 1		Experiment 2	
Group	Young	Old	Young	Old
Age	25.03 ± 4.2	68.75 ± 8.3	21.8 ± 3.1	66.3 ± 5.5
Gender	M = 14, F = 16	M = 17, F = 23	M = 9, F = 11	M = 5, F = 15
Education years	17.2 ± 2.7	14.26 ± 3.3	15.15 ± 1.9	13.95 ± 2.8
MoCa	NA	27.18 ± 1.1	NA	27.75 ± 1.5

Education years: Experiment 1, young versus old: t(68) = 3.92, *p* < 0.001, Cohen’s d = 0.95; Experiment 2, young versus old: t(38) = 1.58, *p* = 0.12, Cohen’s d = 0.5. MoCa: Experiment 1 versus Experiment 2: t(58) = − 0.16, *p* = 0.88, Cohen’s d = − 0.04

#### Task

Participants performed a reinforcement learning task (Fig. 1). An image appeared in the center of the screen together with four gray disks below. Participants were instructed to choose and click on one of the disks. Clicking on a disk led to the presentation of the “next” image, with each of the four disks linked to a different subsequent image. The experiment resembles the above navigation task for finding food, where each decision (e.g., clicking on the right disk) leads to new images until a goal image is reached. In this respect, images correspond to states in RL terminology, and clicking on a disk corresponds to actions. The entire set of images/states constitutes an environment determined by a transition matrix that connects images/states via actions/clicks (Fig. 1B). During navigation through this environment, an “old” image could reoccur multiple times, resulting in participants navigating in a loop. When revisiting an old image, the grey disks led to the same next images as before. For example, whenever the tree image appeared, clicking on the left disk led always to the presentation of the image of a chair. Hence, it is crucial to learn the disk-next-image associations. There was no time limit for choosing a disk. The objective was to find a goal image, labeled with the word “Yeah!”. Before the start of the experiment, all nine possible images were presented on the screen, except for the goal image, which was not presented, but participants were informed about it. Participants initiated the experiment by clicking on a gray disk at the bottom of the screen. An episode consisted of the navigation process until the goal state was found. Participants started the next episode at a randomized location that was at least 2 steps away from the goal state. Furthermore, all these initial states were identical for all the participants.

We used two inter-stimulus intervals (ISI) of 0.5 s (short ISI condition) and 6 s (long ISI condition) between the participant’s responses and the next state, respectively. The objective of the task was to reach the goal state as frequently as possible within 8 min for the short ISI condition, and 30 min for the long ISI condition. The order of the two ISI conditions was counterbalanced among the participants, i.e., half began with the short ISI condition and the other half started with the long ISI condition. There is one transition matrix for each ISI condition, which was identical for all participants.

### Experiment 2

#### Participants

Twenty older healthy adults and twenty young healthy adults were recruited from the Free University of Tbilisi, Georgia. Individual who had previously taken part in the first experiment were no eligible. Detailed demographical information is presented in Table 1.

#### Task

Experiment 2 follows a similar procedure as experiment 1, with the difference that, instead of a fixed duration of 8 min, the number of trials was fixed at 150 for each of the ISI condition. Accordingly, the objective of the task was to reach the goal state as many times as possible within the given number of trials. Furthermore, the order of the two ISI conditions was fixed, with the long ISI condition always coming after the short ISI condition.

Immediately after the RL task, a memory task was given to the participants. In total, there were eighteen images. Twelve of these images were the same as in the RL task, the other six images were novel. For each of the images, participants were asked (1) to indicate whether the given image appeared in the RL task, and (2) to rate their confidence in question one, on a 4-Likert scale.

### Behavioral analysis

#### Data pre-processing

In experiment 1, participants were instructed to reach the goal state as often as possible within 8 min. Due to the nature of the task, different observers visited a different number of images ranging from 30 to 200 due to differences in decision-making and reaction times. To ensure the comparability of the data among the participants, only the first 58 trials (the fifth percentile of the average number of trials among all participants) were used. Any trials exceeding this threshold were discarded. Four participants from the older population were removed from the study, as their total number of trials was lower than 59 trials. It is important to note that this pre-processing procedure only applied to experiment 1 as in experiment 2 the number of trials was fixed for all participants.

#### Behavioral performance

We determined the number of episodes completed and the proportion of optimal actions taken. The number of episodes completed refers to the number of times the participants reached the goal state. The proportion of optimal actions is the number of times a participant chose the that optimally reduced the distance to the goal state from the current state divided by the total number of actions performed in the task. To establish a performance baseline, we simulated an agent performing the task 1000 times, making completely random decisions. At each state, the agent chose an action with uniform probability. The baseline was determined by the average number of episodes completed and the proportion of optimal actions.

Furthermore, we assessed their improvement in performance by calculating the difference in the last and first 29 actions for the number of episodes completed and the proportion of optimal actions. A larger difference indicates better improvement, thereby implying enhanced learning. These four measures are our primary measures. Please note, these measures are not independent of each other. To gain further insights we used a number of secondary measures that are also not independent from the primary measures and can be seen as sub-measures.

First, the nine states of the environment were further categorized into adjacent and distant states. Adjacent states are states that were only one step away from the goal state. The six distant states were at least two steps away from the goal state. We analyzed performance separately for the two measures.

Second, to gain more insight into how performance progressed over time, we calculated the cumulative probability of optimal action across all trials. For each participant, we fitted a log function to estimate the intercept and the slope of the performance curve:

$$y=a+b*log \left(x\right)$$

$x$ represents trial numbers, $a$ and $b$ are the intercept and the slope, respectively, $y$ is the cumulative proportion of optimal actions in each trial.

Third, we tested perseveration behavior. It is essential to efficiently select the optimal actions for a given state in order to achieve superior performance. Choosing repeatedly a non-optimal action in a given is suboptimal and may be related to perseverative behavior or memory deficits. We determined perseverations in two ways. First, we determined the average length of repeating action sequences. We averaged the length of these repetitions of actions across all episodes for each participant. For instance, an episode with the actions “1,2,3,1,2,3,4,2,1” has an average perseveration of two because the action sequence “1,2,3” appeared twice. Second, we calculated the proportion of repeated state-action pairs. In an episode, we extracted all the states and the corresponding actions to determine the probability that the same state-action pair has been visited within the same episode. In order to be considered optimal, a state should not be visited multiple times within an episode.

Fourth we determined the action entropy, measuring the randomness of the actions chosen. The theoretical max entropy is

$${E}_{max}= -\sum ({p}_{max, j}*{log}_{2}{p}_{max, j})$$

The probability distribution of completing each of the four actions was then determined for each state. The entropy of each state is

$${E}_{i}= -\sum ({p}_{(i,j)}*{log}_{2}{p}_{(i,j)})/ {E}_{max}$$

Here,* i* represents states, ranging from one to nine, while *j* represents actions, ranging from one to four. Averaging the action entropy across all states was computed for each participant. High action entropies indicate poor action choices (Sojitra et al. 2018).

Furthermore, we calculated the occupancy map for each state-action pair. Each cell in the matrices represents the number of times a participant engaged with a given state-action pair. Thus, a higher number in a cell indicates more frequent engagement with that specific state-action pair by the participant. Consequently, each participant will have two maps: one for the short ISI condition and one for the long ISI condition. To assess the similarity between these two maps for each participant, we used the cosine distance, calculated as follows:

$${\text{Cosine}}\;{\text{distance}} = {1} - \frac{{\mathop \sum \nolimits_{i = 1}^{n} A_{i} B_{i} }}{{\sqrt {\mathop \sum \nolimits_{i = 1}^{n} A_{i}^{2} } *\sqrt {\mathop \sum \nolimits_{i = 1}^{n} B_{i}^{2} } }}$$

where $A_{i}$ and $B_{i}$ are the elements of vectors A and B, respectively, with each element corresponding to the occupancy counts for a specific state-action pair. Given that there are nine different states and four possible actions from each state, the total number of unique state-action pairs is 36.

#### Computational modelling

A Q-learning model (Sutton and Barto 2018) was used to quantify learning:

$$\left(S,A\right)\leftarrow Q\left(S,A\right)+\alpha \left(\Delta \right)$$

*S* represents the state of the current trial, *A* is the action taken in the current trial, $\Delta$ is the prediction error, *Q* is the Q-value for the given state and action, and $\alpha$ is the free parameter of the learning rate. Whenever a participant performed a new action, the Q value was updated by the prediction error, which is defined as the difference between the current reward and the expected reward:

$$\Delta = r+\underset{i}{\text{max}}Q\left({S}_{new},{A}_{i}\right)-Q\left(S,A\right)$$

*r* stands for the reward, ${S}_{New}$ is the new state after performing action *A* in the given state *S.* The probability of choosing an action is then determined by a soft-max rule:

$$q\left(S,{A}_{i}\right)=\frac{{e}^{\frac{Q\left(S,{A}_{i}\right)}{\tau }}}{{\sum }_{j}{e}^{\frac{Q\left(S,{A}_{j}\right)}{\tau }}}$$

*q* is the probability of choosing action *A* given state *S.* $\tau$ is the free parameter normally referred to as inverse temperature which corresponds to the exploration rate.

#### Statistical analysis

We conducted a two-by-two repeated measures ANOVA with age group (old and young) and ISI (short and long) as independent variables. We quantified the relationship between measurements using Pearson correlations, except for the relationship between measurements and Q-learning model parameters, which were calculated with Spearman’s correlation due to the non-normal distribution of the parameters.

The primary parameters, focusing on performance, learning, and Q-learning model in RL, are presented in the main text. The secondary parameters, are found in the supplementary figures. All details are summarized in Tables S1, S2, and S3.

## Results

### Experiment 1

**Fig. 2:**
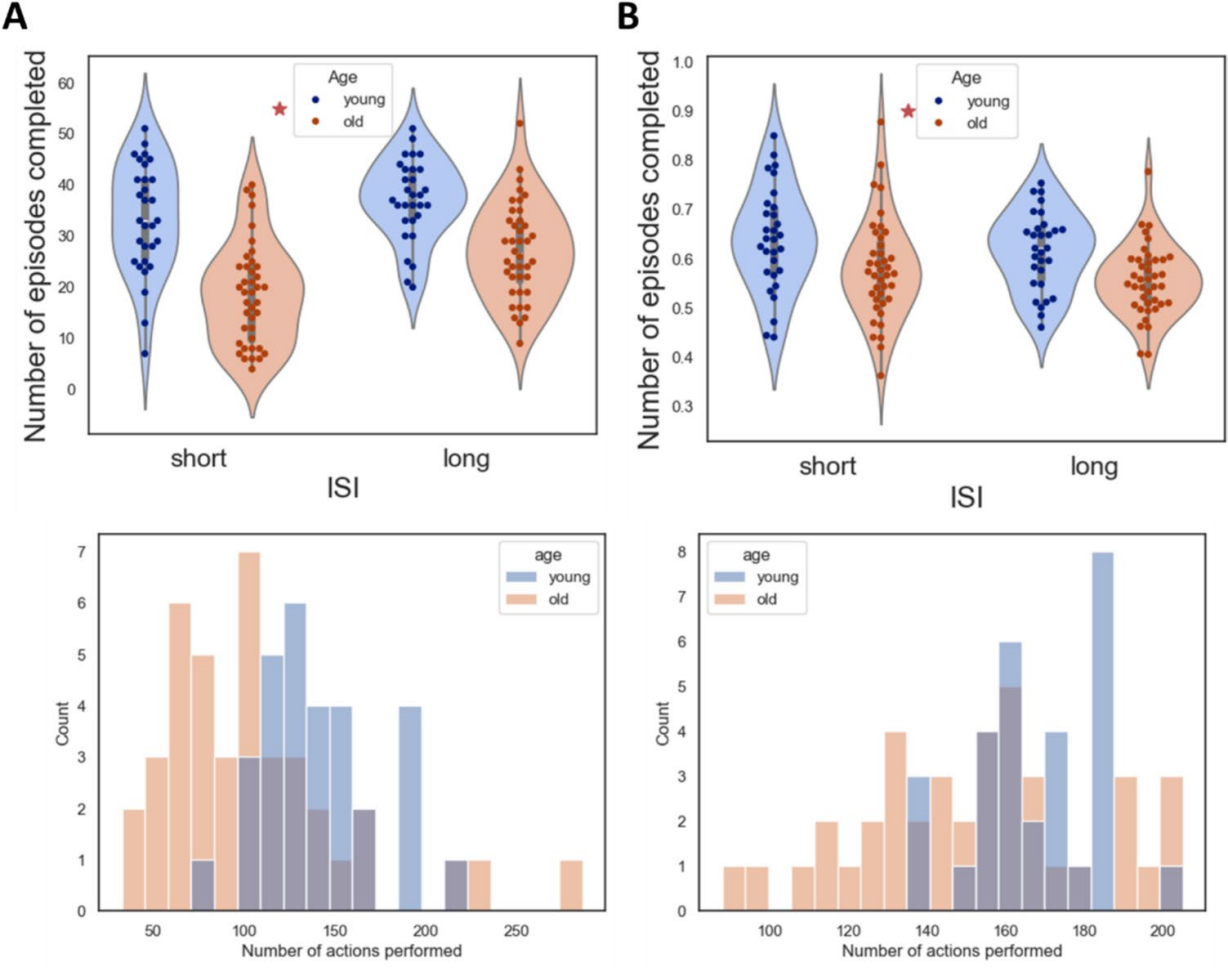
Performance in the navigation task. **A** The number of episodes completed in each ISI condition for each age group. Dots indicate the performance of individual participants. **B** The proportion of optimal actions in each ISI condition for each age group. Red stars indicate a significant difference between the two age groups. **C** Left: The distribution of the number of trials performed for each age groups in the short ISI condition. Right: The same distribution for the long ISI condition

*Effects of ISI and age.* First, we examined the extent to which performance is affected by age. We observed significantly better performance in younger adults compared to older adults (Fig. 2A, F(1,68) = 42.1, *p* < 0.001, partial η^2^ = 0.38; Fig. 2B, F(1,68) = 12.18, *p* < 0.001, partial η^2^ = 0.15). Furthermore, reaction times differed significantly between the two age groups (F(1,68) = 26.17, *p* < 0.001, partial η^2^ = 0.28), with younger adults responding much faster. However, the number of trials completed by the two age groups also differed significantly (Fig. 2C; Kolmogorov–Smirnov test, KS_old versus young (short ISI)_ = 0.52, *p* < 0.001; KS_old versus young (long ISI)_ = 0.38, *p* = 0.009). To account for this bias, we analyzed only the first 58 trials from each participant.

**Fig. 3:**
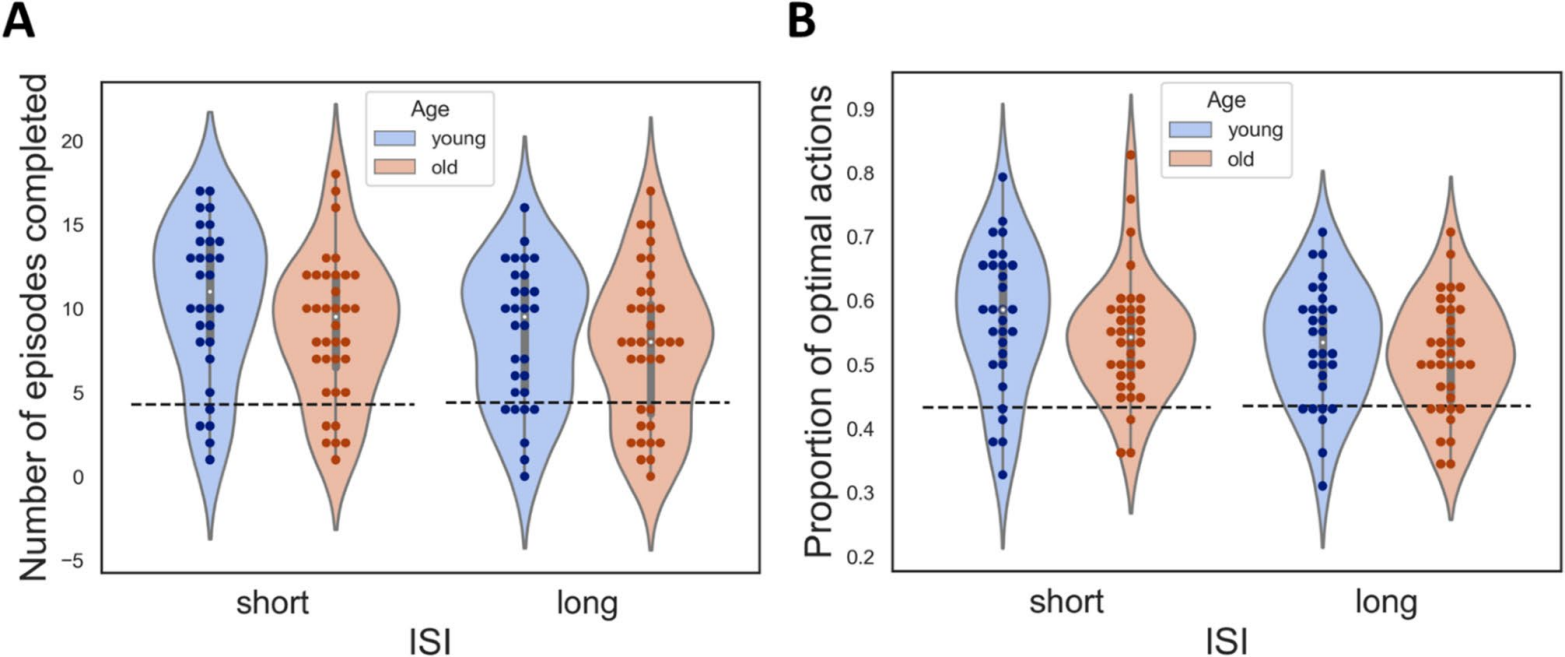
Performance in the RL task, with the first 58 trials. **A** The number of episodes completed in each ISI condition for each age group. Dots indicate the performance of individual participants. **B** The proportion of optimal actions in each ISI condition for each age group. The dashed lines in the figures indicate baseline performance

By equating the number of trials, the number of episodes completed (Fig. 3A) and the proportion of optimal actions (Fig. 3B) did not differ significantly between young and old adults.

#### Improvement of performance

**Fig. 4:**
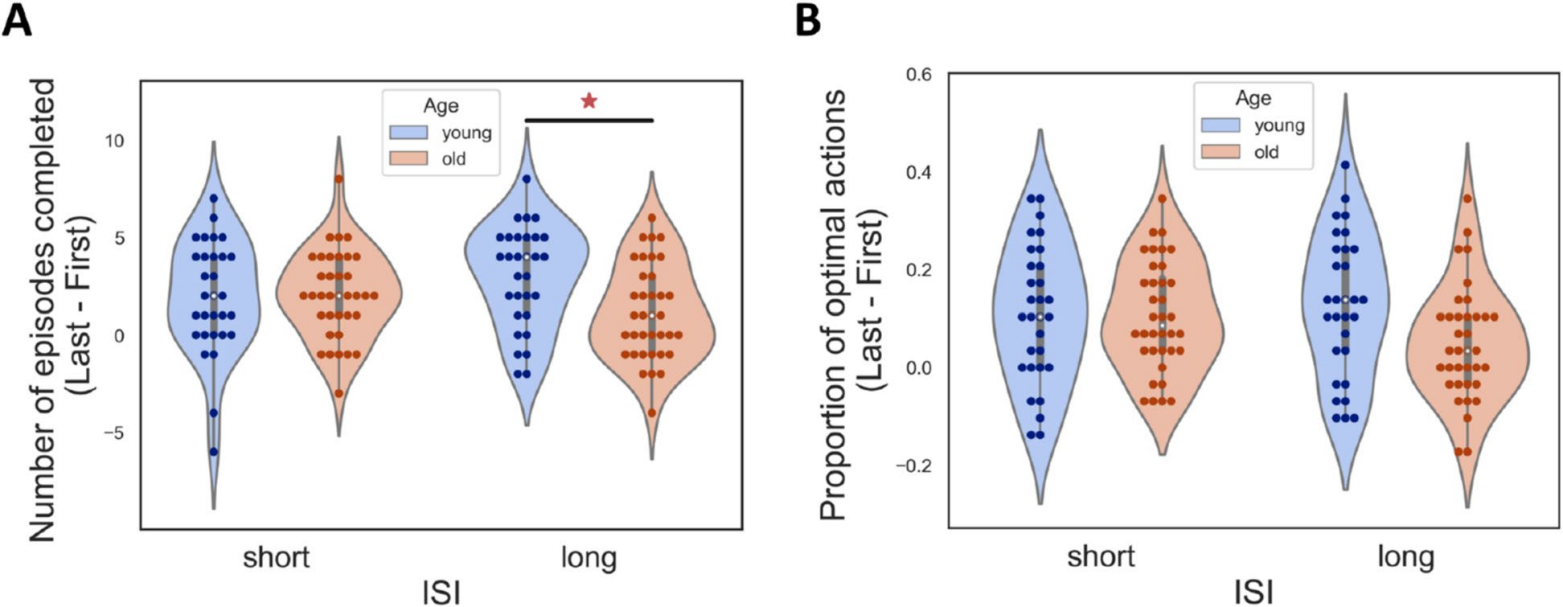
Improvement of performance. The improvement in the accuracy for each age group and ISI condition. **A** The improvement in number of episodes. *p < 0.05 by post-hoc Tukey’s test. **B** Improvement in proportion of optimal actions

We next investigated the improvement of performance during the RL task by dividing the trials into two sets, the first 29 trials and the last 29 trials of each observer. There was a significant interaction between the two groups and ISI when it came to the number of episodes completed. A post-hoc test showed that the long ISI condition drove the interaction (Fig. 4A). A slight trend but no statistically significant improvement in performance was observed (Fig. 4B). For a closer examination, we fitted the accuracy data across trials with a log function which yielded two parameters, the slope and intercept. Both the intercept and the slope did not differ between the groups (Fig. S2A).

#### Perseveration behavior and action entropy

We assessed the extent of suboptimal action selection by quantifying the action entropy, which is a measure of the randomness of actions. Older adults had a lower improvement in action entropy compared to young adults (Fig. S2B). We suggest below that the difference may be caused by memory deficits.

Additionally, we also examined the perseveration behavior, which is characterized by a persistent repetition of suboptimal actions. There were no significant differences between young and older adults (Fig. S2C, left; Fig. S2C, middle). Furthermore, the improvement in perseveration behavior was not significant across the age groups (Fig. S2C, right).

Our findings indicate a difference in the improvement of action entropy between age groups, with the older group showing less improvement compared to the younger group, regardless of the ISI condition.

#### Q-learning

Both learning rates and the inverse temperature do not show significant differences between the two age groups. The learning rate reflects the speed of learning, and the inverse temperature reflects the randomness of choices or exploration rate. The results suggest that both groups revealed similar learning efficiency and exploration through the RL environment.

Effect size and statistical values can be found in Table S1.

### Experiment 2

Given the impact of the number of trials in the first experiment, we conducted a second experiment in which all participants completed 150 trials. This approach makes the comparison and interpretation of the results more comparable and provides additional data to examine whether even more trials lead to performance differences between the two age groups.

#### Effects of ISI and age

**Fig. 5:**
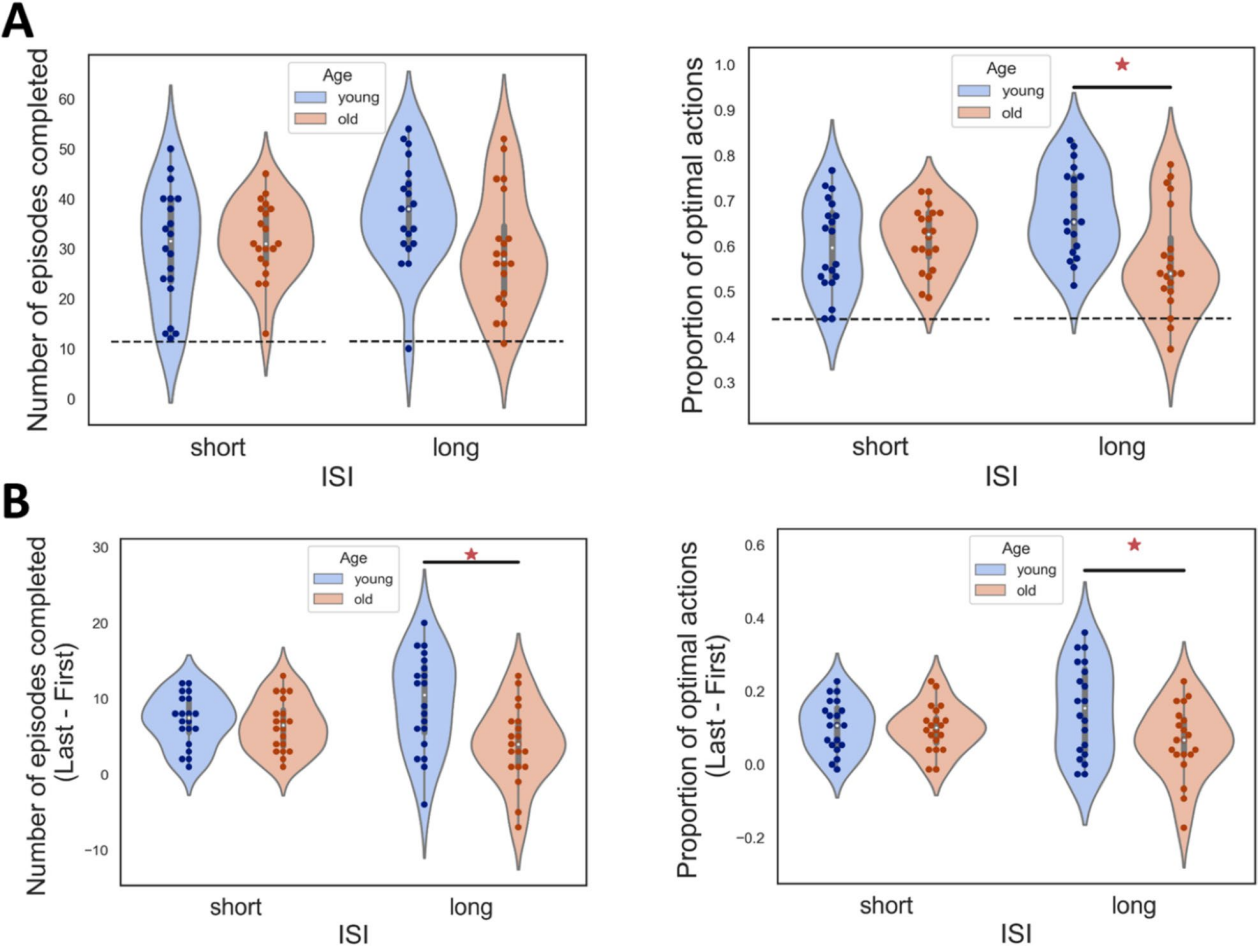
Performance in experiment 2. The conventions are the same as Fig. 2. **A** Left: The number of episodes completed in each ISI condition for each age group. Right: The proportion of optimal action in each ISI condition for each age group. *p < 0.05 post-hoc Tukey’s test. The dashed lines in the figures indicate baseline performance. **B** The improvement in the accuracy for each age group and ISI condition. Left: The improvement in number of episodes. Right: Improvement in proportion of optimal actions. *p < 0.05 by post-hoc Tukey’s test

Contrary to experiment 1, a significant interaction of ISI and age was observed in the proportion of optimal actions (Fig. 5A, right). This interaction effect showed that young adults performed better in the long ISI condition but not in the short ISI condition. Additionally, there was a marginal difference in the number of episodes completed (Fig. 5A, left). Moreover, there was a significant difference in improvement in performance in the long ISI between older and young adults (Fig. 5B), following the same pattern, where young adults performed better only in the long ISI condition.

The results indicate that only in the long ISI condition older and younger adults performed differently. We did not find significant differences in the behavioral patterns between the two groups, suggesting that similar strategies are used to perform the RL task (Fig. S1B, left, t(38) = 1.19, *p* = 0.241, Cohen’s d = 0.38). Furthermore, we observed that the levels of similarity correlate with performance in both older and young participants. Hence, the measurements capture individual differences in task accuracy (Fig. S1B, right r_young_(18) = 0.772, r_old_(18) = 0.731).

#### Questionnaire

The results of the questionnaire revealed that both older and young adults were able to accurately recognize the images used in the RL task, with no participants making mistakes. Next, we measured the confidence rating of their answers. The confidence ratings were based on a scale where a rating of 1 represented high confidence and a rating of 4 indicated high uncertainty. Interestingly, there was no significant difference in the confidence ratings between older and young adults in the adjacent states (Fig. S3B, left). However, a clear main effect of age was observed in the distant states (Fig. S3B, right), with young adults showing higher confidence ratings compared to older adults.

#### Q-learning

**Fig. 6:**
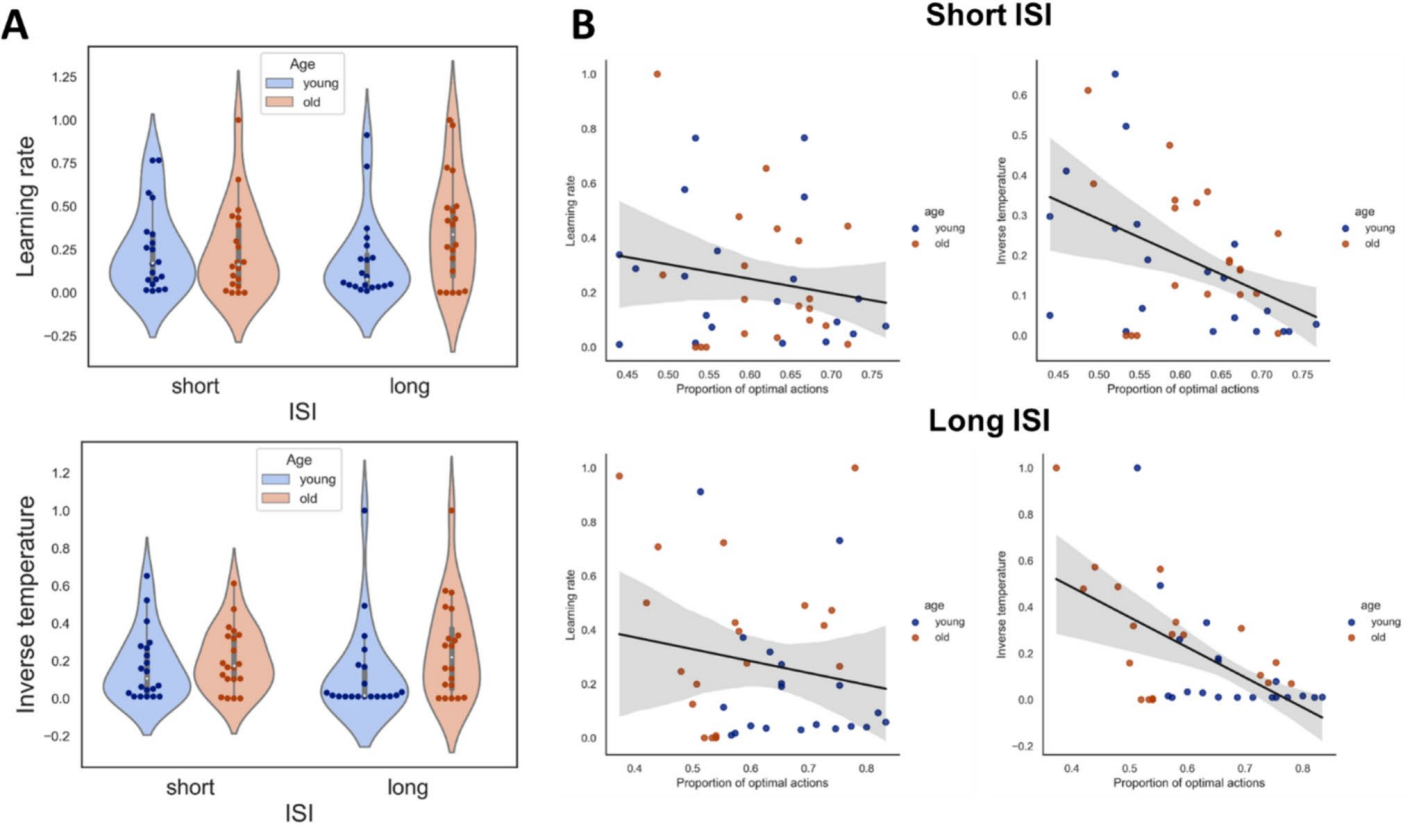
Q-learning model and the performance—experiment 2. The conventions are the same as in Fig. 4. A Q-learning model was fitted to the data and two parameters retrieved from the model for each condition and for each age group: (**A**) Top: The learning rate; Bottom: The inverse temperature. (**B**) The correlation between the parameters and the proportion of optimal actions. Top panel: The correlation between the proportion of optimal actions and the learning rate (left) and the inverse temperature (right) in the short ISI condition. Bottom panel: The correlation between the proportion of optimal actions and the learning rate (left) and the inverse temperature (right) in the long ISI condition

Similar to the results of experiment 1, both learning rates (Fig. 6A, top) and the inverse temperature (Fig. 6A, bottom) did not differ between the two age groups. This suggests that, while there may be some subtle differences in the learning processes of older and younger adults, overall there is not significant difference in the parameters used for Q-learning. However, despite the absence of group differences, the inverse temperature was found to be significantly correlated with accuracy (Fig. 6B, Top right) rather than learning rate (Fig. 6B, Top left). This indicates that while the learning rate may not have a direct effect on performance, the inverse temperature, which controls the exploration–exploitation trade-off, does affect the accuracy.

Effect size and statistical values can be found in Table S2.

*Reaction time.* We observed a significant main effect of age on reaction time in both experiments, with young adults responding faster than older adults (experiment 1: F(1,64) = 27.4, *p* < 0.001, partial η^2^ = 0.3; experiment 2: F(1,64) = 17.33, *p* < 0.001, partial η^2^ = 0.31). To link reaction times with accuracy, we calculated an inverse efficiency score, defined as reaction time divided by the proportion of optimal actions (our measure of accuracy). Consistently, young adults exhibited significantly lower inverse efficiency scores compared to older adults (experiment 1: F(1,64) = 27.4, *p* < 0.001, partial η^2^ = 0.3; experiment 2: F(1,64) = 18.22, *p* < 0.001, partial η^2^ = 0.32). These results indicate that young adults process information more efficiently than older adults, suggesting superior cognitive abilities in complex environments.

## Discussion

In the current study, we investigated age-related changes in RL. In experiment 1, older adults performed as well as young adults with the number of episodes completed, proportion of optimal actions, improvement in proportion of optimal actions, learning rate and the exploration rate. In experiment 2, in which the number of trials was fixed at 150, we found significant age differences in the long ISI condition for improvement in accuracy and the secondary measures derived from this measure. Overall, the results of our study are consistent with previous studies that found intact RL in older adults (Lighthall et al. 2018; Pietschmann et al. 2011).

Moderate to large age differences were observed in the measurements reflecting the performance differences between the two age groups in the long ISI condition. There are several explanations: a genuine RL deficit, a working memory deficit, or stronger fatigue. First, none of the parameters of the Q-learning model was abnormal in the older participants, including learning and exploration rates, which speaks rather against an RL deficit.

Second, we did not observe significant age differences in the short ISI conditions, indicating that working memory is not the key factor underlying any differences in performance. However, in the memory questionnaire, older adults demonstrated less confidence in recognizing distant states. In addition, the improvement of action entropy was less pronounced. These findings may speak indeed suggest a slight deficit at the episodic memory level. Lighthall et al. (2018), employed a RL task also with short and long ISIs. Although the authors did not observe behavioral differences between the two age groups for either ISI condition, they found a significant difference in the hippocampal activity pattern between the age groups in the long ISI condition. Therefore, the higher memory load in the long ISI condition potentially leads to differences in hippocampal activation, but does not manifest at the behavioral level because of the simpler task. Furthermore, we did not observe significant differences in the similarity of occupancy maps across the two ISI conditions between the age groups, indicating that both groups performed in a similar manner. More interestingly, this measure can capture individual differences in accuracy.

Third, it is well known that older people fatigue more quickly than younger ones (Enoka and Duchateau 2016). Hence, instead of memory load, higher fatigue in the older population may explain the age difference in the long ISI condition. However, our analysis only found age difference in the distant states regarding the proportion of optimal actions, indicating that older adults were still able to locate the correct actions when the goal state was close enough. Therefore, the most likely explanation for the age difference seems to be memory-related.

Daniel et al. (2020) suggested that older adults perform worse than younger adults in demanding but not simpler tasks. They had an easy and a harder condition and found no performance differences in the easy condition but a trend for a group comparison in the hard condition. However, the results hint at a ceiling effect in the easy condition (performance between younger and older controls: 97% vs. 94%), which suggests the possibility that a group difference in the easy condition could not be detected due to the task demand. Since task demand does not seem to be the crucial aspect, there must be other reasons for the differences in the paradigms.

One of the limitations related to research on ageing is the large variability in the group of older adults. In addition, the mean age is often different. Small sample sizes may lead naturally to sampling biases, which may lead to different outcomes. In addition, there are demoscopical, socio-economical, and cultural differences. Hence, it may simply be the case that our and others null results come from a too “healthy” and/or still too “young” population (our mean age was 68 and 66 years and in Daniel et al. (2020) it was 70 years). Thus, not the paradigms and differences between paradigms are key, but rather the sampling of the population.

In experiment 1, limiting the analysis to the first 58 trials may seem restrictive. For this reason, we compared the first 29 trials with the last 29 trials and observed similar patterns, except for differences in the long ISI condition. In addition, we conducted experiment 2, where all participants completed the same number of trials. Here, young adults showed improved performance in the later session, but only under the long ISI condition, partially contradicting the hypothesis of consistently superior performance by young adults. Additionally, participants were not instructed to respond quickly, and older adults may have adopted slower response strategies. Hence, the slower response time in older adults does not necessarily indicate a deficit in RL. However, the effect indeed indicates that older adults process information in a slower and more inefficient manner compared to the younger adults. With the current design, we were not able to disentangle if this differences come from slower motor abilities or from higher cogntive processing. We suspect the former since older participants did not reveal a performance difference when controlling for the number of trials. Further research is needed.

Next to performance, learning and exploration rate, we also tested for perseverations, a behavioral pattern where individuals continue to perform repeated action sequences regardless of whether they are optimal or not. We did not find an increased perseveration rate in older adults, contrary to previous results, for example, in the Wisconsin Card Sorting test (Shaqiri et al. 2019). With the very same paradigm used in this study, we tested schizophrenia patients and found evidence for perseverations compared to age-matched controls. Hence, our paradigm is sensitive to perseverations. Perseveration of schizophrenia patients are often attributed to abnormal dopamine levels leading to suboptimal action selection (Durstewitz and Seamans 2008). In RL models, dopamine levels are classically related to the learning rate (Sutton and Barto 2018) and prediction error (Schultz et al. 1997; Wise and Rompre 1989). If this were all true, our results indicate an intact dopaminergic system.

In summary, our findings indicate that reinforcement learning is relatively preserved in older adults, at least when sufficiently many trials are presented. However, for fewer trials, there are deficits. This holds true for a large range of outcome measures including learning and exploration rates in classic Q-learning models.

## Supplementary Information

Below is the link to the electronic supplementary material.

Supplementary file1 (DOCX 1120 KB)

## Data Availability

The data and materials for the experiments reported here are available upon request.

## Code availability

The code for the experiments reported here are available upon request.
