# Supplementary material for: Intact reinforcement learning in healthy ageing
Source: Exp Brain Res. 2025 Jul 11;243(8):185. doi: 10.1007/s00221-025-07092-x (PMC12254157; doi:10.1007/s00221-025-07092-x)
Supplement: Supplementary file 1 — Supplementary file1 (DOCX 1120 KB) [file 221_2025_7092_MOESM1_ESM.docx]

# *Supplementary Information*

| 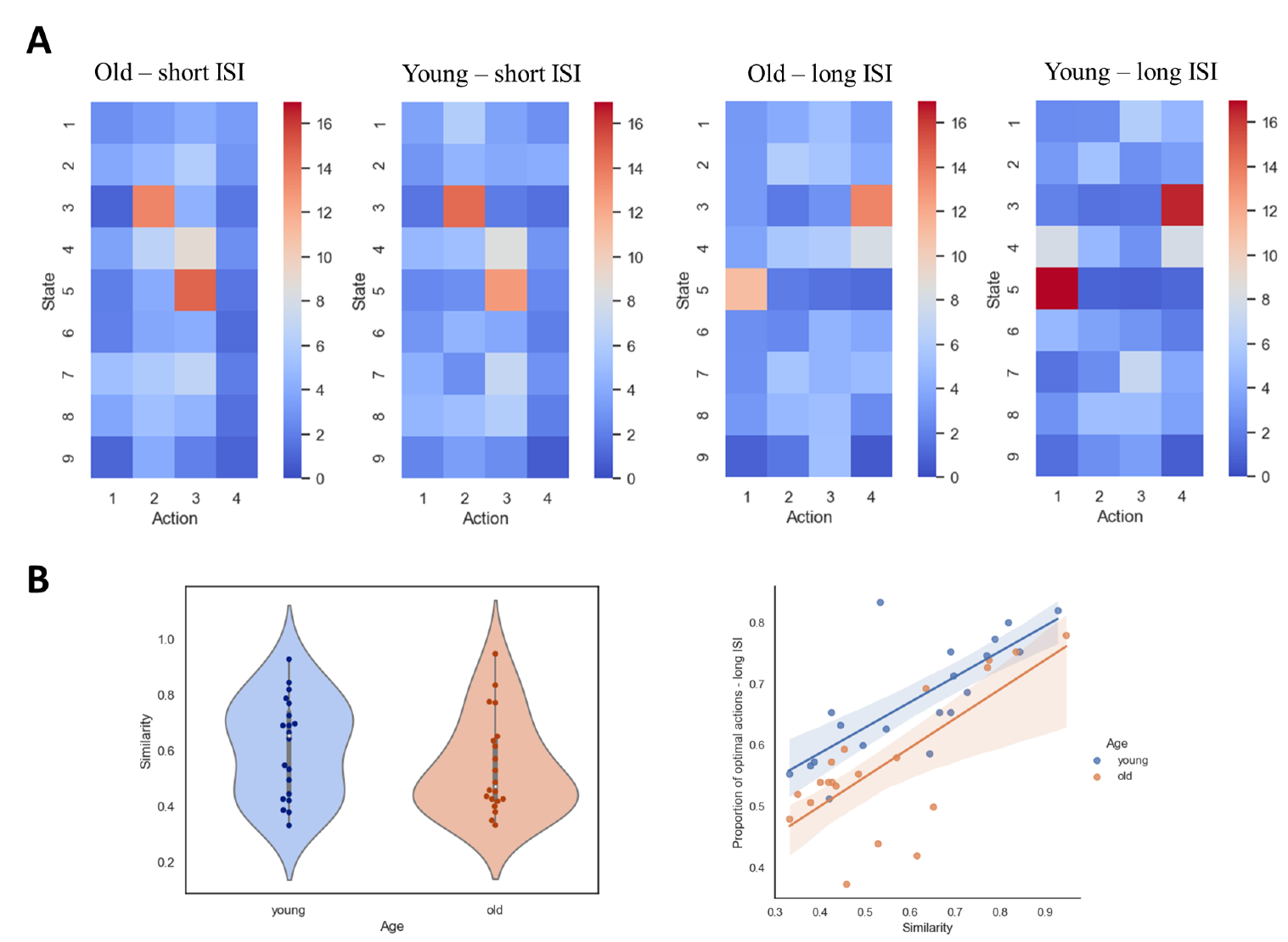 |
| --- |

**Figure S1.** **The** **similarity between the two ISI conditions predicts RL performance.** (A) The average occupancy map for both age groups. The values in each cell represent the average counts for performing specific state-action pairs. (B) Left: The similarity of the maps between the two ISI conditions for each age group. Right: The correlation between the proportion of optimal actions and the levels of similarity.

| 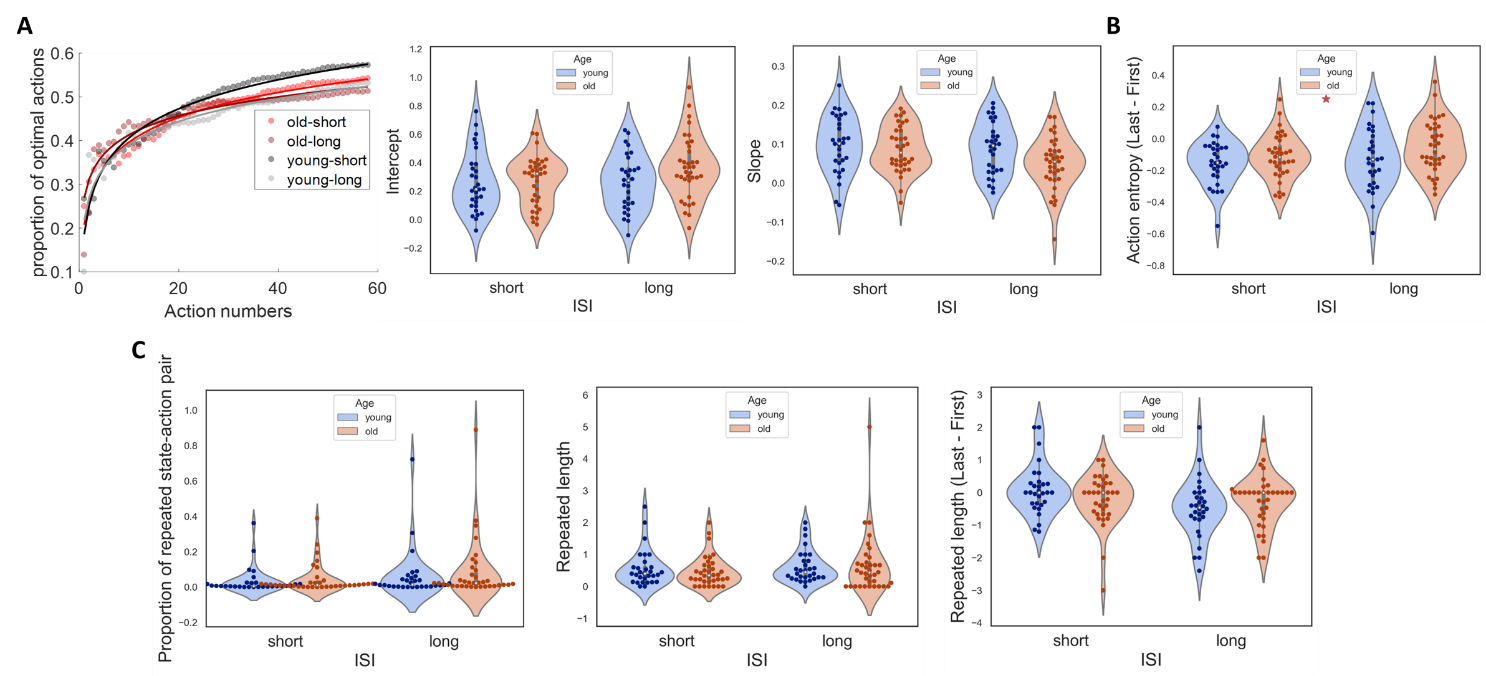 |
| --- |

**Figure S2.** **Perseveration behavior and action entropy.** (A) Left: The cumulative proportion of optimal actions across time. The two ISI conditions and age groups were plotted separately. Each dot represents the average cumulative proportion of optimal actions across the participants in each age group. The solid line shows the fit of the log function. Right: The two bar graphs depict the slopes and intercepts of the fitted log function. (B) The improvement in action entropy. A main effect of age is presented. *: p < 0.05. (C) Left: The proportion of repeated state-action pair of each age group in each ISI condition. Middle: The repeated action length of each age group in each ISI condition. Right: The improvement in the repeated action length.

| 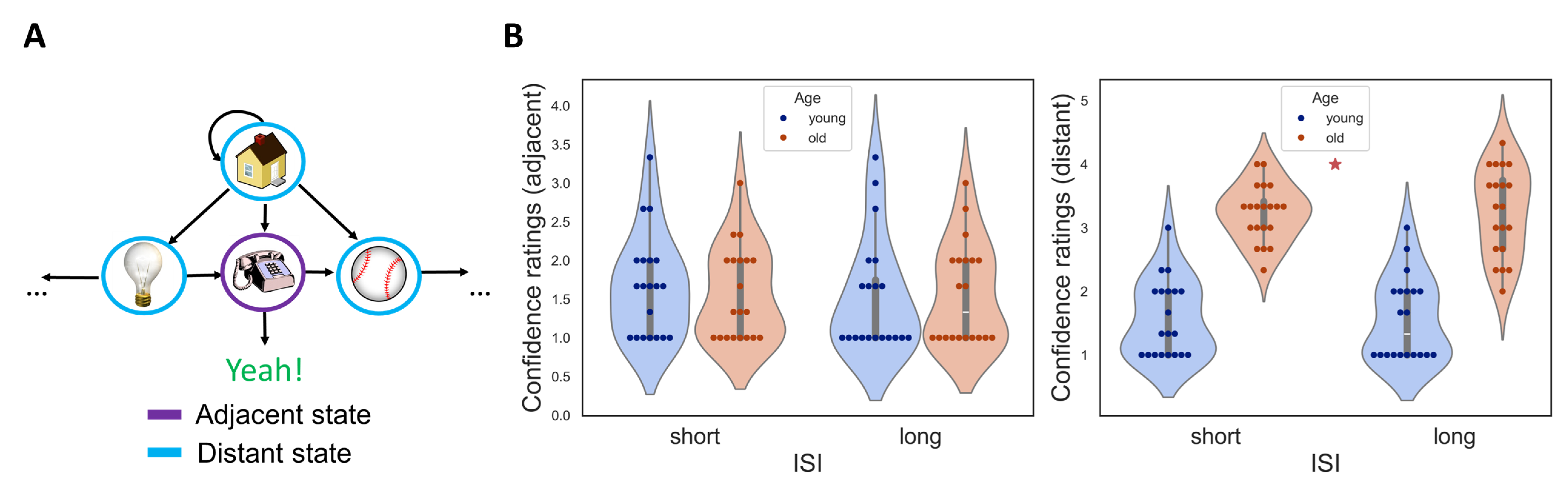 |
| --- |

**Figure S3.** **Secondary measurements for experiment 2**. (A) The adjacent state is indicated in purple, and refers to a state that is directly connected to the goal state. Distant states, indicated in blue, are those that require multiple steps to reach to the goal state. (B) Confidence ratings of the questionnaire. Left: The average confidence ratings in adjacent states for each age group in each ISI condition. Right: The average confidence ratings in distant states for each age group in each ISI condition.

| **Primary parameters – experiment 1** | **Short ISI** | **Long ISI** |
| --- | --- | --- |
| Number of episodes completed (3A) | age: F(1,64) = 1.98, p = 0.16, partial η^2^ = 0.03  interaction: F(1,64) = 0.44, p = 0.51, partial η^2^ = 0.007 | |
| Proportion of optimal actions (3B) | age: F(1,64) = 1.83, p = 0.18, partial η^2^ = 0.03  interaction: F(1,64) = 0.14, p = 0.71, partial η^2^ = 0.002 | |
| Improvement in number of episodes completed (4A) | Age: F(1,64) = 3.66, p = 0.06, partial η^2^ = 0.054  interaction: F(1,64) = 5.4, **p = 0.02**, partial η^2^ = 0.078 | |
|  | ns | post-hoc test **p_tukey_ = 0.018**  Cohen’s d = 0.74 |
| Improvement in proportion of optimal actions (4B) | age: F(1,64) = 3, p = 0.09, partial η2 = 0.045  interaction: F(1,64) = 3.1, p = 0.08, partial η2 = 0.046 | |
| Q-learning-alpha (full-trial) | age: F(1,68) = 3.5, p = 0.07, partial η^2^ = 0.05  interaction: F(1,68) = 0.04, p = 0.85, partial η^2^ = 0 | |
| Q-learning-tau (full-trial) | age: F(1,68) = 9.77, **p = 0.003**, partial η^2^ = 0.13  interaction: F(1,68) = 0.06, p = 0.8, partial η^2^ = 0.001 | |
| Q-learning-alpha | age: F(1,64) = 0.39, p = 0.54, partial η^2^ = 0.006  interaction: F(1,64) = 1.77, p = 0.19, partial η^2^ = 0.027 | |
| Q-learning-tau | age: F(1,64) = 2.98, p = 0.09, partial η^2^ = 0.044  interaction: F(1,64) = 0.07, p = 0.79, partial η^2^ = 0.001 | |
| **Secondary parameters – experiment 1** |  |  |
| Intercept of log fitting (S2A) | F(1,64) = 1.89, p = 0.17, partial η^2^ = 0.03 | |
| Slope of log fitting (S2A) | F(1,64) = 1.89, p = 0.18, partial η^2^ = 0.03 | |
| Improvement in action entropy (S2B) | age: F(1,64) = 5.21, **p = 0.026**, partial η^2^ = 0.075;  interaction: F(1,64) = 0.37, p = 0.55, partial η^2^ = 0.006 | |
| Proportion of repeated-action pair (S2C) | age: F(1,64) = 0.59, p = 0.45, partial η^2^ = 0.009  interaction: F(1,64) = 0.09, p = 0.77, partial η^2^ = 0.001 | |
| Repeated action length (S2C) | age: F(1,64) = 0.06, p = 0.81, partial η^2^ = 0.001  interaction: F(1,64) = 0.39, p = 0.53, partial η^2^ = 0.006 | |

**Table S1.** **Summary of parameters for experiment 1.**

| **Primary parameters – experiment 2** | **Short ISI** | **Long ISI** |
| --- | --- | --- |
| Number of episodes completed (5A) | age: F(1,38) = 1.55, p = 0.22, partial η^2^ = 0.04  interaction: F(1,38) = 4.84, **p = 0.03**, partial η^2^ = 0.11 | |
|  | Ns | ns |
| Proportion of optimal actions (5A) | age: F(1,38) = 3.55, p = 0.07, partial η^2^ = 0.08  interaction: F(1,38) = 9.77, **p = 0.003**, partial η^2^ = 0.2 | |
|  | Ns | post-hoc test **p_tukey_ = 0.005**  Cohen’s d = -1.1 |
| Improvement in number of episodes completed (5B) | age: F(1,38) = 10.1, **p = 0.003**, partial η^2^ = 0.21  interaction: F(1,38) = 4.37, **p = 0.043**, partial η^2^ = 0.1 | |
|  | Ns | post-hoc test **p_tukey_ = 0.003**  Cohen’s d = -1.14 |
| Improvement in proportion of optimal actions (5B) | age: F(1,38) = 5.1, p = 0.03, partial η^2^ = 0.12  interaction: F(1,38) = 4, p = 0.052, partial η^2^ = 0.095 | |
|  | Ns | post-hoc test **p_tukey_ = 0.018** Cohen’s d = -0.95 |
| Q-learning-alpha (6A) | age: F(1,38) = 1.88, p = 0.18, partial η^2^ = 0.05  interaction: F(1,38) = 2.4, p = 0.13, partial η^2^ = 0.06 | |
| Q-learning-tau (6A) | age: F(1,38) = 2.71, p = 0.11, partial η^2^ = 0.07  interaction: F(1,38) = 0.81, p = 0.37, partial η^2^ = 0.02 | |
| **Secondary parameters – experiment 2** |  |  |
| Memory questionnaire – adjacent (S3) | age: F(1,38) = 0.03, p = 0.86, partial η^2^ = 0.0008 interaction: F(1,38) = 0.6, p = 0.44, partial η^2^ = 0.016 | |
| Memory questionnaire – distant (S3) | age: F(1,38) = 110.37, **p < 0.001**, partial η^2^ = 0.74 interaction: F(1,38) = 0.006, p = 0.94, partial η^2^ = 0.0004 | |

**Table S2.** **Summary of parameters for experiment 2.**

| \| **Parameter 1** \| **Parameter 2** \| **Short ISI** \| **Long ISI** \| \| --- \| --- \| --- \| --- \| \| Improvement in number of episodes completed \| Q-learning alpha \| ns \| r(64) = 0.4, p < 0.001 \| \| Improvement in number of episodes completed \| Q-learning tau \| r(64) = -0.4, p < 0.001 \| ns \| \| Proportion of optimal actions \| Q-learning alpha (6B) \| ns \| ns \| \| Proportion of optimal actions \| Q-learning tau (6B) \| r(38) = -0.41, p = 0.009 \| r(38) = -0.35, p = 0.029 \| |
| --- | --- | --- | --- | --- | --- | --- | --- | --- | --- | --- | --- | --- | --- | --- | --- | --- | --- | --- | --- | --- |

**Table S3.** **Summary of all correlation measurements.**
